# Intestinal and neuronal myenteric adaptations in the small intestine induced by a high-fat diet in mice

**DOI:** 10.1186/s12876-015-0228-z

**Published:** 2015-01-22

**Authors:** Angelica Soares, Evandro José Beraldi, Paulo Emílio Botura Ferreira, Roberto Barbosa Bazotte, Nilza Cristina Buttow

**Affiliations:** 1 Center of Medical and Pharmaceutical Sciences, State University of the West of Paraná, R. Universitária, 1619, Cascavel, PR CEP 85819-110 Brazil; 2 Department of Morphological Sciences, State University of Maringá, Av. Colombo, 5790, Maringá, PR CEP 87020-900 Brazil; 3 Department of Pharmacology and Therapeutics, State University of Maringá, Av. Colombo, 5790, Maringá, PR CEP 87020-900 Brazil

**Keywords:** Intestinal wall, Myenteric plexus, Myosin-V, Neuronal nitric oxide synthase, Vasoactive intestinal peptide, Enteroendocrine cells, Goblet cells, Intraepithelial lymphocytes

## Abstract

**Background:**

The prevalence of obesity has increased at alarming rates, particularly because of the increased consumption of high-fat diets (HFDs). The influence of HFDs on intrinsic innervation and the intestinal wall has not been fully characterized. The aim of this study was to investigate the morpho-quantitative aspects of myenteric neurons and the wall of the small intestine in mice fed a HFD.

**Methods:**

Swiss mice were fed a HFD (59% kcal from fat) or standard chow (9% Kcal from fat) for 8 weeks. Segments of the duodenum, jejunum, and ileum were subjected to histological processing for morpho-quantitative examination of the intestinal wall and mucosal cells, and immunohistochemistry was performed to evaluate myenteric neurons. The data for each segment were compared between the groups using an unpaired Student’s *t*-test or an equivalent nonparametric test.

**Results:**

The HFD increased body weight and visceral fat and decreased the length of the small intestine and the circumference of the ileum. In the duodenum, the HFD increased the density of the nitrergic subpopulation and decreased the area of nitrergic neurons and vasoactive intestinal peptide (VIP) varicosities. In the jejunum, the density of the nitrergic subpopulation was increased and the neuronal areas of the general population, nitrergic subpopulation and (VIP) varicosities were reduced. In the ileum, the density of the general population and nitrergic subpopulation were increased and the neuronal areas of the general population, nitrergic subpopulation and (VIP) varicosities were reduced. The morphometric parameters of the villi, crypts, muscular layer and total wall generally increased in the duodenum and jejunum and decreased in the ileum. In the duodenum and jejunum, the HFD promoted a decreased in the proportion of intraepithelial lymphocytes. In the ileum, the proportion of intraepithelial lymphocytes and goblet cells reduced, and the enteroendocrine cells increased.

**Conclusions:**

The high-fat diet induces changes in the myenteric innervation of the small intestine, intestinal wall and mucosal cells responsible for the secretion of hormones and maintenance of the protective intestinal barrier. The morpho-quantitative data provide a basis for further studies to clarify the influence of HFD in the motility, digestive and absorptive capacity, and intestinal barrier.

## Background

Obesity is a challenge for global public health, particularly with regard to the association with chronic diseases, such as diabetes, hypertension, cardiovascular disease, and cancer [1]. According to global data, 14% of adult women and 10% of adult men are obese [1]. Increasing rates of obesity reflect behavioral changes in modern society, including the greater intake of foods that are rich in fat and have a high energy density [2].

The consumption of a high-fat diet (HFD), usually produced with 20-60% fat [3], is associated with the development of obesity in humans [2] and animals [3-5]. In animals, HFDs also induce disorders similar to those that occur in human obesity, such as hyperglycemia [4], insulin resistance, and type 2 diabetes [5].

Some studies have reported the influence of a HFD on the gastrointestinal tract. However, the responses are distinct when different sources of saturated fats are compared (68:28 saturated:monounsaturated fat *vs*. 39:45 saturated:monounsaturated fat) [6,7] and when these are compared with high monounsaturated fats (12:80 saturated:monounsaturated fat) [6]. For example, in the intestinal mucosa, saturated fat (39:45 saturated:monounsaturated) increased villus height in the jejunum and ileum after only 3 days in animals that underwent bowel resection [7], while high saturated fat (68:28 saturated:monounsaturated) for 8.4 weeks reduced villus height in these same segments [6]. Fat also affects mucus secretion, the number of goblet cells [8] and the cell proliferation [7,8]. These changes may influence the nutrient absorption and protective function of the mucosal barrier. With regard to the cell populations of the intestinal epithelium, intraepithelial lymphocytes and the mucus barrier, produced mainly by goblet cells, are related to the immune defense and can be affected by HFDs [9].

Enteroendocrine cells are another cell population that is related to nutrient absorption, which secrete gastrointestinal hormones that work in an integrated manner to optimize digestion and absorption [10]. Furthermore, evidence indicates that these cells also interact with the nervous system, can activate local neural circuits, and promote motor, secretory, and vasodilator activity [11].

Previous studies have reported the influence of fat on gastrointestinal motility [12,13]. The pathogenesis of these alterations might involve changes in intrinsic innervation represented by the enteric nervous system. Recent studies described myenteric neuropathy after HFD consumption [5,14]. Myenteric neurons are differentially affected, and subpopulations of neurons that express neuronal nitric oxide synthase (nNOS) and vasoactive intestinal peptide (VIP) are particularly susceptible [5]. Because of this intimate relationship, myenteric neuroplasticity can be accompanied by structural changes in the muscular tunic and intestinal wall, which has been suggested in the literature [15,16].

The present study evaluated the effects of a HFD on the general neuronal population (myosin-V-immunoreactive [IR]) and nitrergic (nNOS-IR) and VIP-ergic (VIP-IR) subpopulations in the myenteric plexus of the small intestine of mice. Additionally, the morphology of the intestinal wall, cell proliferation, and subpopulations of goblet cells, enteroendocrine cells, and intraepithelial lymphocytes were studied.

## Methods

### Animals and study groups

Male Swiss mice (*Mus musculus*) were obtained from the Central Biotherium of the State University of Maringá. The animals were maintained under a controlled room temperature (22 ± 2°C) and 12 h/12 h light/dark cycle and housed in separate cages. All of the procedures were approved by the Ethics Committee on Animal Experiments at the State University of Maringá and followed international laws for animal protection.

At 42 days of age, the animals weighed an average of 34 g were distributed into two groups with 10 animals each: control animals (CON group) that were fed standard rodent chow (9% kcal from fat) (Nuvilab, Quimtia SA, Colombo, PR, Brazil) and experimental animals that were fed a HFD that contained 59% kcal from fat for 8 weeks (OB group). The HFD (Table 1) was prepared using lard in a composition that was exactly the same as previously used by Arçari *et al.* [4] and based on the AIN-93G purified diet [17]. Lard has approximately 40:40 saturated:monounsaturated fat and is rich in palmitic acid, a long-chain triglyceride. The amount of fatty acids in the standard chow was 4% of the total weight; in the HFD, it was 35%. Both groups were allowed access to food and water *ad libitum*.

**Table 1:** **Nutritional composition of the standard chow and high-fat diet (HFD)**

	Standard chow	HFD
	g/100 g	
Protein	22	20
Carbohydrate	55	35
Total fat	4	35
Fiber	7	5
Micronutrients	12	5
kcal/kg	3773	5358

### Intestine collection

After fasting for 15 h, the animals received 0.5 mg/kg of vincristine sulfate solution 2 h prior to euthanasia (Tecnocris, Eurofarma, São Paulo, SP, Brazil) intraperitoneally to block mitosis in the intestinal epithelium. The animals were weighed and intraperitoneally anesthetized with 80 mg/kg Thiopental (Abbott Laboratories, Chicago, IL, USA) and underwent laparotomy to collect the small intestine and periepididymal, retroperitoneal, and mesenteric fat deposits. The fat deposits were weighed, and the lengths (from the pylori to the ileo-cecal junction) of the small intestine were measured. Samples of the duodenum, jejunum, and ileum were opened at the mesenteric border immediately after collection and the intestinal circumference was measured. From the 10 animals in each group, five underwent immunohistochemical procedures, and the other five underwent histological procedures.

### Immunohistochemical techniques

Samples of the duodenum, jejunum, and ileum were washed with phosphate-buffered saline (PBS; 0.1 M, pH 7.4), tied at both ends, filled in and distended with 4% buffered paraformaldehyde (pH 7.4) for 2 h. After fixation, the samples were opened at the mesenteric border, washed with PBS, and microdissected under a stereomicroscope. The mucosa and submucosa tunics were removed, and the muscle layer was retained to obtain whole-mounts of the muscular tunic that contained the myenteric plexus.

The whole-mounts were subjected to immunohistochemical techniques for the observation of the general population of myosin-V-IR myenteric neurons, subpopulation of nNOS-IR myenteric neurons, and varicosities of nerve fibers of VIP-IR myenteric neurons distributed throughout the circular muscle.

The whole-mounts were washed twice in PBS with 0.5% Triton-X100 (PBS-T) and incubated in blocking solution that consisted of PBS-T, 2% bovine serum albumin (BSA), and 10% non-immune goat serum for 1 h at room temperature. After blocking, the whole-mounts were incubated with anti-myosin-V [18], anti-nNOS, or anti-VIP primary antibody (Table 2) in an incubation solution of PBS-T that contained 2% BSA and 2% goat serum for 48 h at room temperature with shaking. The whole-mounts were washed three times in PBS-T and incubated for 2 h at room temperature in an incubation solution that contained the secondary antibody (Table 2) with shaking. They were then washed three times in PBS-T and mounted on glass slides with 10% PBS in glycerol.

**Table 2:** **Primary and secondary antibodies used in immunoreactions for myosin-V, nNOS and VIP**

Antibody	Host	Dilution	Company
Myosin-V (Primary)	Rabbit	1:200	Buttow *et al.* [18]
nNOS (Primary)	Rabbit	1:500	Zymed
VIP (Primary)	Rabbit	1:500	Península Laboratories, Inc.
Anti-rabbit IgG FITC (Secundary)	Rabbit	1:500	Santa Cruz Biotecnology

### Quantitative and morphometric analysis of immunoreactive myenteric neurons

The analyses were performed in images captured with an AxioCam MRC high-resolution camera (Carl Zeiss, Jena, Germany) coupled to an Axioshop Plus fluorescence microscope (Carl Zeiss, Jena, Germany) at 200× (myosin-V-IR and nNOS-IR neurons) and 400× (VIP-IR varicosities) magnification. The images were transferred to a computer using Axio Vision Rel software (v. 4.6) and analyzed using Image Pro Plus software (v. 4.5, Media Cybernetics, Silver Spring, MD, USA).

The images were captured by randomly sampling across all of the whole-mounts on the histological slides, with no specific visual fields chosen, and the same field was not captured more than once. Immunoreactive neurons (myosin-V-IR and nNOS-IR) that were present in 30 images per animal were counted for each segment. The area of each image was approximately 0.36 mm^2^, and the total quantified area was 10.93 mm^2^. The results are expressed as neurons per cm^2^.

The areas of 100 neuronal cell bodies (myosin-V-IR and nNOS-IR) per animal were measured for each segment, for a total of 500 neurons in each segment per group. Measurements were made in neurons where it was possible to clearly see the limits of the cell body. For each animal, the areas of 400 varicosities that were found in the circular muscle (VIP-IR) were measured per animal in each segment, for a total of 2,000 per group; overlapping varicosities were not measured. The results are expressed in μm^2^.

### Morphometric analysis of the intestinal wall

Samples of the duodenum, jejunum, and ileum were opened at the mesenteric border, washed with saline solution, fixed in Bouin’s solution for 6 h, dehydrated in alcohol, diaphanized in xylol, and embedded in paraffin. Semi-serial longitudinal sections (28 μm intervals, 4 μm thick) were subjected to hematoxylin-eosin (HE) staining.

To evaluate villus height, crypt depth, and muscular layer and intestinal wall thickness (from the villus apex to the mesothelium of the tunica serosa), 40 measurements per animal were randomly made by a blind observer for each variable in each segment using Image Pro Plus 4.5 image analysis software (Media Cybernetics, Silver Spring, MD, USA). The digital images were captured with a high-resolution camera (Q Color 3 Olympus American, Burnaby, BC, Canada) coupled to a microscope (Olympus BX 41, Olympus, Tokyo, Japan) using Q Capture Pro 5.1 software.

### Quantitative analysis of cell proliferation, goblet and enteroendocrine cells, and intraepithelial lymphocytes

The analyses were performed with semi-serial histological sections that were stained using the following methods. The HE method was used to quantify cell proliferation using the metaphasic index and quantify intraepithelial lymphocytes (IELs). The histochemical periodic acid-Schiff (PAS) technique was used to quantify goblet cells that contained neutral mucins [19]. The histochemical Grimelius technique that consists of impregnation by silver [20] was used to quantify enteroendocrine cells.

Goblet cells and IELs were quantified throughout the villi. The number of goblet cells or IELs from one side of the villus and total cell number on the same side were counted for a total of approximately 2,500 cells for each technique per segment in each animal. The number of enteroendocrine cells was similarly counted in the crypt-villus axis for a total of approximately 2,500 cells per segment in each animal.

The metaphasic index was determined by counting the number of cells at metaphase from one side of the crypts and total number of crypt cells for a total of approximately 2,500 cells per animal for each segment.

The quantifications were performed using a Zeiss Primo Star microscope (Carl Zeiss, Jena, Germany) at 400× magnification. The data are reported as the number of specific cell populations per total number of cells multiplied by 100.

### Statistical analysis

The data were tested for a normal distribution and for each parameter assessed. The group data for each individual segment were compared using an unpaired Student’s *t*-test or equivalent nonparametric Mann–Whitney U test using Prism 5.01 software (GraphPad, San Diego, CA, USA). Values of *p* < 0.05 were considered statistically significant.

## Results

### Obesity assessment and intestinal dimensions

After 8 weeks of HFD consumption (59% kcal from fat), the animals exhibited a 14% increase (*p* < 0.05) in body weight and 144% increase (*p* < 0.0001) in visceral fat weight (Table 3). The HFD promoted a 10% reduction of the small intestine length and 40% reduction of the ileum circumference (*p* < 0.05; Table 3).

**Table 3:** **Parameters assessed in the control group (CON group) and high-fat diet fed group (OB group)**

	CON	OB
Body weight (g)	41.7 ± 1.3	47.7 ± 1.6*^a^
Visceral fat (g)	1.6 ± 0.2	3.9 ± 0.3***^a^
Length of the small intestine (cm)	56.8 ± 1.8	51.2 ± 1.3*^a^
Circumference of the duodenum (cm)	0.6 ± 0.02	0.5 ± 0.05^b^
Circumference of the jejunum (cm)	0.5 ± 0.02	0.5 ± 0.0002^b^
Circumference of the ileum (cm)	0.5 ± 0.02	0.3 ± 0.02*^b^

Results are expressed as the mean ± SEM (n = 10). *p < 0.05, ***p < 0.0001 vs CON group; ^a^unpaired *t*-test; ^b^nonparametric test.

### Neuronal morphology and density

**Figure 1:**
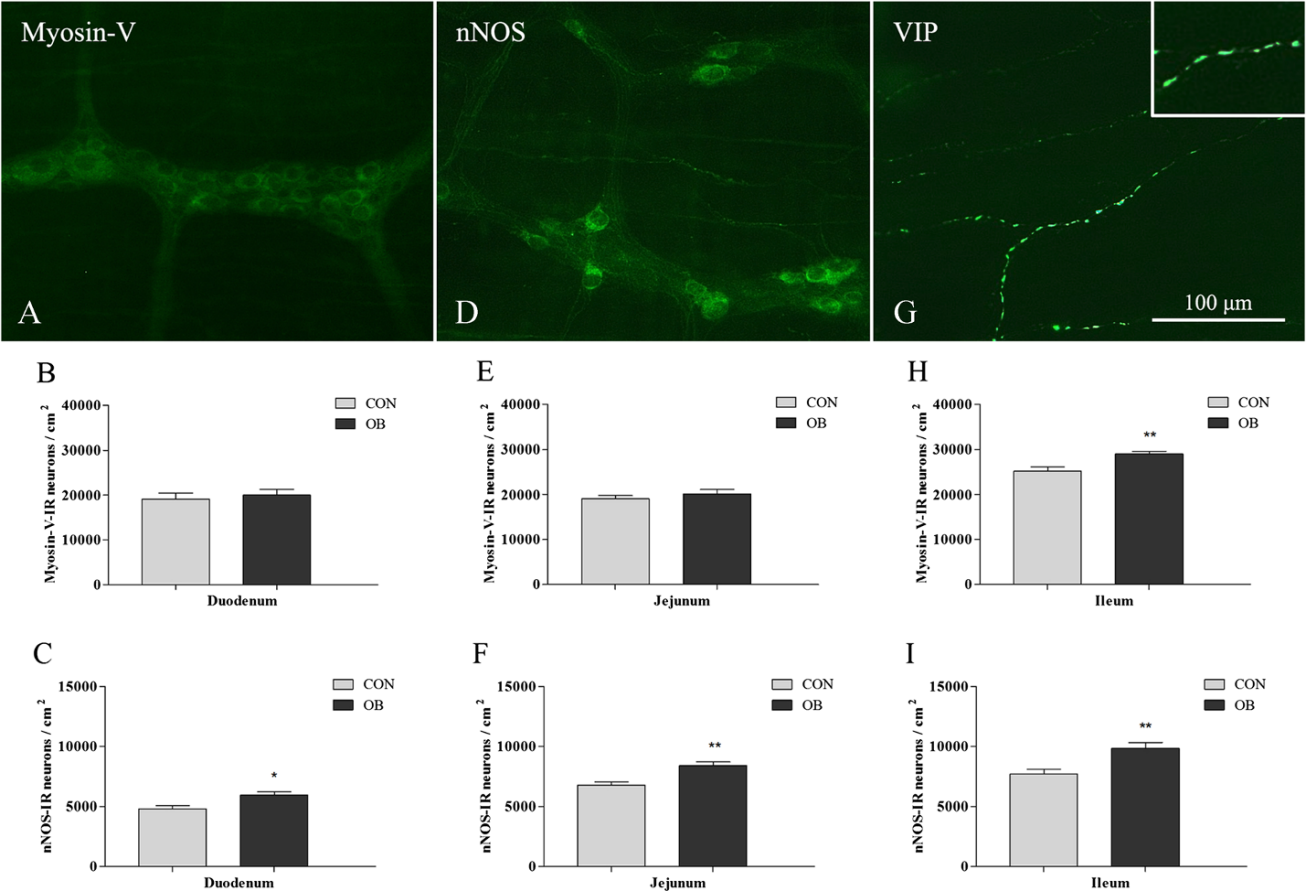
**Neurons of the myenteric plexus and neuronal density in the small intestine of mice.** Representative micrographs of neurons in the ileum of the control group, showing the myosin-V-IR general population **(A)**, nNOS-IR subpopulation **(D)** and VIP-IR varicosities **(G)** (insert: varicosities detail). Quantitative analysis (neurons per cm^2^) of myosin-V-IR myenteric neurons and nNOS-IR neurons between groups CON and OB in the duodenum **(B, C)**, jejunum **(E, F)** and ileum **(H, I)**. The HFD increased the density of myosin-V-IR neurons in the ileum and nNOS-IR neurons in all segments of the small intestine. Results are expressed as mean ± SEM (n = 5); *p < 0.05, **p < 0.01 vs CON group in each intestinal segment; unpaired *t*-test.

Regardless of the diet, the general organization of the myenteric plexus was unchanged. Myosin-V-IR neurons of different sizes were arranged mainly within the ganglia and rarely along the nerve fibers (Figure 1). The subpopulation of nitrergic neurons was located mainly peripherally in the ganglion (Figure 1).

In the duodenum, no difference in neuronal density of the general population (myosin-V-IR) was observed between the CON and OB groups (*p* > 0.05; Figure 1). With regard to the nitrergic subpopulation, the HFD increased the density of nNOS-IR neurons in the OB group (23%, *p* < 0.05; Figure 1).

In the jejunum, the neuronal density of the myosin-V-IR population was also unchanged (*p* > 0.05) between the CON and OB groups, but an increase in the nNOS-IR subpopulation (24%, *p* < 0.01) was observed in the OB group compared with the CON group (Figure 1).

The HFD significantly increased the neuronal density of the myosin-V-IR population (16%, *p* < 0.01) and nNOS-IR subpopulation (28%, *p* < 0.01) in the ileum of the OB group (Figure 1).

### Neuronal morphometry

**Figure 2:**
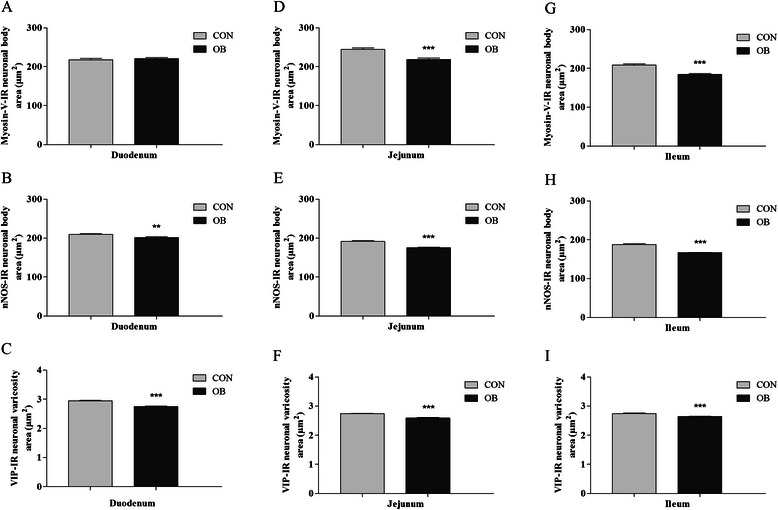
**Myenteric neuronal morphometry in the small intestine of mice.** Morphometric analyses of myosin-V-IR neuronal cell bodies areas, nNOS-IR cell bodies areas and VIP-IR varicosities between groups CON and OB in the duodenum **(A-C)**, jejunum **(D-F)** and ileum **(G-I)**. The HFD reduced the area of myosin-V-IR neuronal cell bodies (except in the duodenum), nNOS-IR cell bodies and VIP-IR varicosities in all segments of the small intestine. Results are expressed as mean ± SEM (n = 5); **p < 0.01, ***p < 0.0001 vs CON group in each intestinal segment; nonparametric test.

In the duodenum of mice fed the HFD, no changes were observed in the size of neuronal cell bodies of the myosin-V-IR general population compared with the CON group. The HFD reduced the areas of cell bodies of then NOS-IR subpopulation (4%, *p* < 0.01) and reduced the areas of VIP-IR myenteric varicosities (7%, *p* < 0.0001) in the OB group (Figure 2).

In the jejunum, the areas of myosin-V-IR neuronal cell bodies (11%, *p* < 0.0001), nNOS-IR neuronal cell bodies (9%, *p* < 0.0001) and VIP-IR varicosities (5%, *p* < 0.0001) were reduced in the OB group compared with the CON group (Figure 2).

The HFD also decreased the size of myosin-V-IR neuronal cell bodies (12%, *p* < 0.0001), nNOS-IR neuronal cell bodies (12%, *p* < 0.0001) and VIP-IR varicosities (4%, *p* < 0.0001) in the ileum in the OB group (Figure 2).

### Small intestine morphometry

The morphometric parameters of the intestinal wall are presented in Table 4. In the duodenum, villus height (*p* < 0.0001), crypt depth (*p* < 0.05), muscular layer thickness (*p* < 0.0001), and wall thickness (*p* < 0.0001) increased in the OB group. In the jejunum, villus height increased (*p* < 0.0001), crypt depth decreased (*p* < 0.05), and total wall thickness increased (*p* < 0.01). In the ileum, all of the parameters decreased with the HFD (*p* < 0.0001).

**Table 4:** **Intestinal parameters in the control group (CON group) and high-fat diet fed group (OB group)**

	Duodenum		Jejunum		Ileum	
	CON	OB	CON	OB	CON	OB
Villi height (μm)	307.1 ± 4.9	353.9 ± 4.3***^b^	267.9 ± 4.1	311.4 ± 2.6***^b^	199.8 ± 2.5	169.7 ± 1.5***^b^
Crypts depth (μm)	90.6 ± 0.9	95.4 ± 1.3^b^	89.0 ± 0.8	87.2 ± 0.8*^b^	90.2 ± 0.9	67.6 ± 0.6***^a^
Muscular layer (μm)	37.7 ± 0.5	42.0 ± 0.5***^b^	35.3 ± 0.4	35.7 ± 0.3^a^	50.9 ± 0.6	41.6 ± 0.4***^b^
Total wall (μm)	438.1 ± 5.8	486.9 ± 6.5***^b^	404.2 ± 4.9	421.6 ± 3.5**^a^	355.7 ± 4.3	299.1 ± 2.1***^b^
Cell proliferation (%)	15.9 ± 0.5	16.6 ± 1.6^a^	15.6 ± 1.0	13.0 ± 1.7^a^	15.4 ± 0.5	15.1 ± 1.2^a^
Enteroendocrine cells (%)	0.5 ± 0.1	0.5 ± 0.1^a^	0.5 ± 0.1	0.5 ± 0.1^a^	0.4 ± 0.05	0.6 ± 0.03*^a^
Goblet cells (%)	5.1 ± 0.3	5.0 ± 0.4^a^	6.2 ± 0.6	7.4 ± 0.4^a^	17.0 ± 0.8	14.0 ± 0.4**^a^
Intraepithelial lymphocytes (%)	6.5 ± 0.1	6.1 ± 0.1**^a^	7.9 ± 0.1	6.6 ± 0.1***^a^	11.0 ± 0.1	7.3 ± 0.3***^a^

Results are expressed as the mean ± SEM (n = 5). *p < 0.05, **p < 0.01, ***p < 0.0001 vs CON group in each intestinal segment; ^a^unpaired *t*-test; ^b^nonparametric test.

### Cell proliferation, goblet and enteroendocrine cells, and IELs

The intestinal mucosal epithelium results are presented in Table 4. In the duodenum, the proliferation of mucosal cells in the crypts and the proportion of enteroendocrine and goblet cells were unchanged between the CON and OB groups. The proportion of IELs was reduced in the OB group compared with the CON group (6%, *p* < 0.01).

Similarly in the jejunum, the proportion of cell proliferation, enteroendocrine and goblet cells were unchanged between the CON and OB groups, and the IELs proportion was reduced in the OB group (16%, *p* < 0.0001).

The cell proliferation was also unchanged in the ileum, but the HFD increased the proportion of enteroendocrine cells (50%, *p* < 0.05), decreased goblet cells (18%, *p* < 0.01) and IELs (34%, *p* < 0.0001) in the OB group, compared with the CON group.

## Discussion

The results showed that the HFD that contained 59% kcal from fat induced obesity and alterations in the myenteric plexus, intestinal wall structure, density of goblet and enteroendocrine cells, and IELs in mucosal cells.

The high lipid content of the HFD simulated a modern diet in humans. The body weight gain and visceral fat accumulation observed in these animals are characteristic of moderate obesity [21].

### High-fat diet and general neuronal myenteric density

The general population of myenteric neurons, reflected by immunohistochemistry for the detection of myosin-V protein, a marker of neuronal cells [22], did not change in the duodenum or jejunum with the HFD. In the ileum, the largest neuronal density observed in the OB group was likely a consequence of decreases in the wall thickness and circumference observed in this segment, promoting a larger neuronal concentration per area, which has been reported in other experimental models [16,23]. Nezami and colleagues observed neuronal loss in the colon of CF1 mice with a diet of 60% kcal from fat for 11 weeks [13]. In C57BL/6 mice, neuronal loss was also observed in the duodenum with a HFD of 72% kcal from fat for 8 weeks [5] and in the ileum and colon with a 45% kcal diet after 6 months [14]. These studies suggest that neuronal loss may vary according to the different levels of lipids in the diets, different intestinal segments, mouse strains, and feeding periods. In the present study, unknown is whether the neuronal density was maintained without neurodegeneration because assessing the total area of the ileum (by measuring its length) was impossible because of the absence of macroscopically visible limits in this segment. Using the same experimental model as in the present study, we previously observed significant reductions of the length and circumference of the large intestine, and we utilized a correction factor to calculate the reduction of the area, indicating neuronal loss [24].

### High-fat diet increases nitrergic neuronal density

Although the general neuronal population was maintained, the HFD influenced the nitrergic subpopulation, causing an increase in the density of nNOS-IR neurons. This increase may indicate a change in the chemical code and consequent changes in intestinal motility, because myenteric neurons of the mouse small intestine that contain nNOS are mostly inhibitory motor neurons (90%) that innervate the muscular layer and also descending interneurons (10%) [25] that are involved in local motility reflexes [26]. This would mainly imply slower intestinal transit and increases in the retention and absorption of nutrients, corroborating the morphometric results obtained from the intestinal villi in the duodenum and jejunum. Supporting this hypothesis, mice that were fed a HFD exhibited a greater gastrointestinal transit time [13].

### High-fat diet reduces the neuronal cell body area and neuronal varicosities

The morphometric analysis also revealed neuroplasticity, which was reflected by a reduction of the cell body areas of myosin-V-IR neurons in the jejunum and ileum and nNOS-IR neurons in the three segments. These alterations could be related to the changes observed in the intestinal wall because the plasticity and survival of autonomic neurons appear to involve interactions between neurons and their targets, associated with the production and responsiveness to target-derived neurotrophic factors and actions of components of the extracellular matrix [27]. Indeed, the influence of fat on intestinal muscular cells was described in a study with cell cultures that were treated with palmitic acid (i.e., a saturated fatty acid) and oleic acid (i.e., an unsaturated fatty acid), which are common in HFDs, both inducing cell loss [14].

The decrease in neuronal size would more likely be a consequence of metabolic and structural derangements caused by the excess of saturated fatty acids in the HFD. Palmitic acid, found in great quantity in the HFD used in this study, promotes mitochondrial dysfunction and oxidative stress [13,14]. Such conditions can cause organelle damage [13] and reduce the availability of energetic substrates, leading to a decrease in metabolic activity [14].

The area of VIP-IR varicosities was also reduced in the OB group. This reduction may be attributable to the low expression of VIP mRNA or low neurotransmitter reserves in varicosities caused by greater VIP release [28]. Nitric oxide (NO) induces the release of VIP in the myenteric plexus [28], and the reduction of varicosities observed in the present study may be attributable to an increase in the production and release of NO due to a higher density of nitrergic neurons (nNOS-IR). The greater release of this neurotransmitter could represent plasticity against the possible deleterious effects of the HFD to maintain neuronal survival. A previous study reported an association between VIP release and myenteric neuroprotection [29].

Alternatively, considering that in the myenteric plexus of the small intestine of mice nNOS and VIP are colocalized in inhibitory motor neurons [25], the observed increase in neuronal density (nNOS-IR) could result in a lower demand for neurotransmitter and lower expression of VIP mRNA. The reduction of the cell body areas of nNOS-IR neurons could also alter the cellular metabolic activity and neurotransmitter synthesis.

Furthermore, intracellular derangements caused by the HFD could affect vesicular transport and neurotransmitter reserves in the varicosities. Using a HFD (72% kcal from fat), Stenkamp-Strahm *et al*. [5] observed axonal swelling, a disruption of the cytoskeletal network of microtubules and neurofilaments, and neuronal loss.

### High-fat diet alters intestinal wall morphometry

The neuronal changes may reflect morphometric alterations in the small intestine, in which we observed decreases in the total length and circumference of the ileum. These changes may be the result of an increase in the absorption of the HFD and/or lower food consumption observed in this model [30].

Interestingly, the observed changes in the length and circumference support the morphometric results found in the wall of the small intestine. The villus height and intestinal wall thickness increased in the duodenum and jejunum and decreased in the ileum in the OB group. The changes suggest adaptations related to the digestibility [31] and consistency [32] of the HFD or an increase in the retention of food in the duodenum and proximal jejunum caused by a decrease in motility in these segments. Previous studies showed that the absorption of nutrients occurs mostly in the proximal intestine, and few nutrients reach the ileum [33]. Furthermore, a HFD reduces the motility of the duodenum [34]. This decrease in motility may have been sustained by the increase in the density of (inhibitory) nitrergic neurons in the present study (Figure 1). As result, the HFD may have influenced intestinal absorption capacity, favoring weight gain and visceral adiposity. The distinct changes in the ileum suggest the participation of other factors, such as the microbiota, that have particular characteristics in the ileum [35] and influence the morphology of villi and crypts [19]. Despite the alterations in the morphometry of the villi, the alterations in the mucosa did not result from changes in the cell proliferation rate.

### High-fat diet influences cell populations of the intestinal mucosa

The alterations in the intestinal wall were also accompanied by changes in specific cellular populations. Regarding enteroendocrine cells, an increase in the proportion of the general population, demonstrated by Grimelius staining, was observed in the ileum in the OB group. The reduction of the intestinal circumference likely did not influence this result because the cell proportion was assessed relative to the number of epithelial cells.

The greater quantity of fat [36-39] and/or nutrients [39-41] that reach the terminal ileum likely induced stem cell differentiation, influencing the hormonal release of peptide YY [36,37], glucagon-like peptide 1 (GLP-1) [38], and GLP-2 [39] by the subpopulation of L cells in this region [36,42,43]. Considering the action of the released hormones, functions related to the slowing of intestinal transit time and motility may have been enhanced [44-46]. According to the literature, the inhibitory response of GLP-1 depends on the production and release of NO [45,46], such that GLP-1 appears to act in parallel to an inhibitory mechanism that depends on NO in the smooth muscle [45]. Altogether, our data suggest an interaction between intrinsic innervation and the intestinal epithelium and support the hypothesis that the HFD reduces intestinal motility.

Unlike enteroendocrine cells, the proportion of goblet cells in the ileum and proportion of IELs in the three segments were reduced by the HFD. Thus, functions related to the formation of the mucosal protective barrier through mucus production [47] and immune responses [48] were influenced by the HFD.

The data reflect the proportion of these cells compared with epithelial cells, reducing the possibility that a reduction of intestinal dimensions promoted lower functional demand, suggesting that the barrier function was compromised. Accordingly, the reduction of the number of goblet cells [49] and deficiency of MUC2 mucin [50] have been associated with altered barrier permeability and intestinal inflammation.

High-fat diets have been associated with changes in the intestinal microbiota [51]. As mentioned above, the microbiota of the ileum is different from other segments, and previous studies have suggested that dietary constituents and fermentation products may regulate the secretory function of goblet cells [19,52]. With regard to the subpopulation of IELs, the HFD in the present study consisted of purified ingredients, including isolated protein, refined sugars, and purified sources of vitamins and minerals [17]. The protein content in diets might influence the number of IELs [53]. The standard chow used in our study had proteins from different vegetal sources, whereas the HFD contained only casein [17], which may had lower influence over this population because its lower antigenicity.

## Conclusions

In conclusion, the HFD containing 59% kcal from fat induced myenteric neuroplasticity and adaptations in the wall and mucosal cells after eight weeks of treatment along the intestinal segments. The decreases in the areas of neuronal cell bodies and varicosities suggest a reduction of metabolic activity that may be associated with cellular injury. Changes in the neuronal chemical code, morphological alterations in the intestinal wall, and a larger representation of cells that secrete gastrointestinal hormones suggest adjustments that may result in a decrease in intestinal motility and increase in digestive and absorptive capacity. Additionally, changes in the proportion of cells of the immune system and mucus secretion may result in a modification of the protective function of the intestinal barrier. These observations may provide important insights into changes in the gastrointestinal tract that may eventually lead to intestinal dysfunction and the development of obesity.

## Abbreviations

BSA: Bovine serum albumin
GLP-1: Glucagon-like peptide 1
GLP-2: Glucagon-like peptide 2
HE: Hematoxylin-eosin
HFD: High-fat diet
IEL: Intraepithelial lymphocyte
IR: Immunoreactive
NO: Nitric oxide
nNOS: Neuronal nitric oxide synthase
PAS: Periodic acid-Schiff
PBS: Phosphate-buffered saline
PBS-T: Phosphate-buffered saline with Triton-X100
PYY: Peptide YY
VIP: Vasoactive intestinal peptide
